# Asymmetric bagging and feature selection for activities prediction of drug molecules

**DOI:** 10.1186/1471-2105-9-S6-S7

**Published:** 2008-05-28

**Authors:** Guo-Zheng Li, Hao-Hua Meng, Wen-Cong Lu, Jack Y Yang, Mary Qu Yang

**Affiliations:** 1 Institute of Systems Biology, Shanghai University, Shanghai 200444, China; 2 School of Computer Engineering & Science, Shanghai University, Shanghai 200072, China; 3 Department of Chemistry, School of Science, Shanghai University, Shanghai 200444, China; 4 Harvard Medical School, Harvard University, Cambridge, Massachusetts 02140-0888 USA; 5 National Human Genome Research Institute National Institutes of Health (NIH) U.S., Department of Health and Human Services Bethesda, MD 20852 USA

## Abstract

**Background:**

Activities of drug molecules can be predicted by QSAR (quantitative structure activity relationship) models, which overcomes the disadvantages of high cost and long cycle by employing the traditional experimental method. With the fact that the number of drug molecules with positive activity is rather fewer than that of negatives, it is important to predict molecular activities considering such an unbalanced situation.

**Results:**

Here, asymmetric bagging and feature selection are introduced into the problem and asymmetric bagging of support vector machines (asBagging) is proposed on predicting drug activities to treat the unbalanced problem. At the same time, the features extracted from the structures of drug molecules affect prediction accuracy of QSAR models. Therefore, a novel algorithm named PRIFEAB is proposed, which applies an embedded feature selection method to remove redundant and irrelevant features for asBagging. Numerical experimental results on a data set of molecular activities show that asBagging improve the AUC and sensitivity values of molecular activities and PRIFEAB with feature selection further helps to improve the prediction ability.

**Conclusion:**

Asymmetric bagging can help to improve prediction accuracy of activities of drug molecules, which can be furthermore improved by performing feature selection to select relevant features from the drug molecules data sets.

## Background

Modeling of quantitative structure activity relationship (QSAR) of drug molecules will help to predict the molecular activities, which reduce the cost of traditional experiments, simultaneously improve the efficiency of drug molecular design [1]. Molecular activity is determined by its structure, so structure parameters are extracted by different methods to build QSAR models. Many machine learning methods have been used to the modeling of QSAR problems, like multiple linear regression, k-nearest neighbor [2], partial least squares [3], Kriging [4], artificial neural networks [5] and support vector machines (SVM), of which SVM is a state-of-arts method and achieved satisfactory results in the previous studies [6-8].

Nowadays, ensemble learning is becoming a hot topic in the machine learning and bioinformatics communities [9], which has been widely used to improve the generalization performance of single learning machines. For ensemble learning, a good ensemble is one whose individuals are both accurate and make their errors on different parts of the input space [9]. The most popular methods for ensembles creation are Bagging and Boosting [10-12]. The effectiveness of such methods comes primarily from the diversity caused by re-sampling the training set. Agrafiotis et al. [13] compared bagging with other single learning machines on handling QSAR problems and found that bagging is not always the best one. Signal was proposed in [14], it created an ensemble of meaningful descriptors chosen from a much larger property space which showed better performance than other methods. Random forest was also used in QSAR problems [15]. Dutta et al. used [16] different learning machines to make an ensemble to build QSAR models, and feature selection is used to produce different subsets for different learning machines.

Although the above learning methods obtained satisfactory results, but most of the previous works ignored a critical problem in the modeling of QSAR that the number of positive examples often greatly fewer than that of negatives. To handle this problem, Hou et al. [17] discussed this problem and assigned different costs for two different classes of SVM and improved the prediction results. Here combing ensemble methods, we propose to use asymmetric bagging of SVM to address the unbalanced problem. Asymmetric bagging of SVM has been used to improve relevance feedback in image retrieval [18]. Instead of re-sampling from the whole data set, asymmetric bagging keeps the positive examples fixed and re-samples only from the negatives to make the data subset of individuals unbalanced. Furthermore, we employ AUC (area under ROC curves) [19] as the measure of predictive results, because only the measure of prediction accuracy of correction can not show the overall performance. We will analysis the experimental results in terms of AUC and other several popular measures like sensitivity and specificity as well as correction. Furthermore, In QSAR problems, many parameters are extracted from the molecular structures as features, but some features are redundant and even irrelevant, these features will hurt the generalization performance of learning machines [20]. For feature selection, different methods can be categorized into the filter model, the wrapper model and the embedded model [20-22], where the filter model is independent of the learning machine and both the embedded model and the wrapper model are depending on the learning machine, but the embedded model has lower computation complexity than the wrapper model has. Different methods have been applied to QSAR problems [23-25], and shown that proper feature selection of molecular descriptor will help improve the prediction accuracy.

In order to improve the accuracy of asymmetric bagging, we will use the feature selection methods to improve the accuracy of individuals, this is motivated by the work of Li and Liu's work [26], where they found embedded feature selection is effective to improve accuracy of bagging of SVM and proposed an algorithm PRIFEB, which improved generalization performance of ordinary bagging. Here we propose to combine PRIFEB with asymmetric bagging and develop a novel algorithm named PRIFEAB to solve the prediction problem of unbalanced QSAR.

## Results and discussion

In order to demonstrate the effect of unbalanced learning methods, we have performed the following series experiments by using support vector machine (SVM) as base classifiers.

1. SVM is a baseline method, which uses a 2-norm soft margin version of SVM.

2. unSVM assigns different *C *for different classes. The parameter of balanced_bridge is set as the value of the ratio of the number of positive examples to that of negatives which is 0.0188 in this paper.

3. Bagging a commonly used ensemble method, which uses SVM as base learners. The number of individuals is 55.

4. unBagging is also a commonly used bagging method, which uses unSVM as base learners. There are also 55 individuals.

5. asBagging is asymmetric bagging which uses SVM as base learners.

6. PPIFEAB is a bagging method, which employs feature section for asBagging to remove irrelevant and redundant features.

### Prediction performance

Experiments are performed to investigate if asymmetric bagging and feature selection help to improve performance of bagging. Support vector machines with *C *= 100, *σ *= 0:1 are used as individual classifiers, and the number of individuals is 55 for all bagging methods. For unSVM, balanced_bridge is used to denote the ratio of *C*_+ _to *C*_-_, which is 0.0188. For ordinary bagging, each individual has one tenth of the training data set, while for asBagging, the size of individual data subset is twice of the positive sample in the whole data set. The 3-fold cross validation scheme is used to validate the results, experiments on each algorithm are repeated 10 times. We test the learning methods on individual molecular descriptors, and there are BCUT, Constitutional, Prop and Topological descriptors, which are represented by BCUT, CONST, PROP and TOPO respectively.

**Figure 1:**
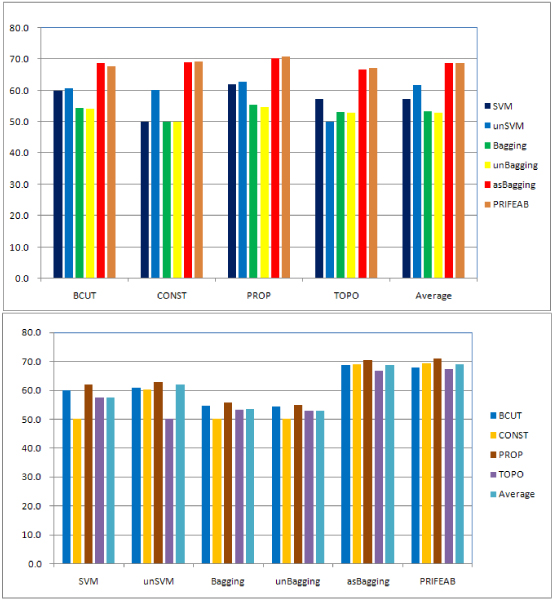
P**erformance of different learning algorithms**. Both graphs show BACC scores. Top: Results grouped by descriptors. Bottom: Results grouped by different learning algorithm.

The average BACC values are shown in Figure 1, from which, we can obviously find that:

(1) unSVM does improve performance of SVM.

(2) Bagging does not reach our expectation, it does not improve performance of SVM, so does unBagging, which has the similar results of Bagging.

(3) asBagging greatly improves performance of SVM, and PRIFEAB slightly improve results of asBagging.

Tables 1, 2, 3, 4, 5, 6, 7 list the results of different measures i.e. AUC, BACC, sensitivity, specificity, PPV, NPV, correction by using the above SVM and bagging methods. We also list the ratio values of the number of features used in PRIFEAB to the total number in Table 8. From tables 1, 2, 3, 4, 5, 6, 7, 8, we can see that:

**Table 1:** Statistics values of AUC (%).

Descriptor	SVM	unSVM	Bagging	unBagging	asBagging	PRIFEAB
BCUT	59.4(1.3)	61.2(1.3)	55.0(1.0)	55.2(1.0)	75.3(0.8)	75.8(0.6)
CONST	50.8(0.8)	59.3(1.9)	50.3(1.1)	50.4(1.1)	75.0(0.3)	75.3(0.5)
PROP	62.3(1.4)	63.0(1.2)	55.4(1.5)	55.5(1.3)	78.0(0.9)	78.3(0.9)
TOPO	57.7(1.0)	50.8(0.8)	54.0(1.1)	54.1(2.0)	73.4(0.5)	73.6(0.7)
Average	57.6(1.1)	58.6(1.3)	53.7(1.2)	53.8(1.4)	75.4(0.6)	75.8(0.7)

Outside of parentheses represent the mean of the respective performance measure, while Inside of parentheses correspond to the standard deviation across the 10 times of 3-fold cross validations.

**Table 2:** Statistics values of BACC (%).

Descriptor	SVM	unSVM	Bagging	unBagging	asBagging	PRIFEAB
BCUT	60.0(0.3)	60.8(0.9)	54.5(1.0)	54.2(1.0)	68.7(0.7)	67.8(0.5)
CONST	50.0(0.1)	60.1(0.6)	50.0(0.1)	50.1(0.3)	69.1(0.4)	69.3(0.5)
PROP	62.0(1.0)	62.8(0.4)	55.6(1.1)	54.7(1.3)	70.3(0.1)	71.0(0.9)
TOPO	57.4(0.5)	50.0(0.0)	53.2(0.1)	52.8(1.0)	66.8(0.4)	67.3(0.8)
Average	57.4(0.5)	58.4(0.5)	53.3(0.6)	53.0(0.9)	68.7(0.4)	68.9(0.7)

Outside of parentheses represent the mean of the respective performance measure, while Inside of parentheses correspond to the standard deviation across the 10 times of 3-fold cross validations.

**Table 3:** Statistics values of sensitivity (%).

Descriptor	SVM	unSVM	Bagging	unBagging	asBagging	PRIFEAB
BCUT	20.4(0.7)	22.3(1.7)	9.8(1.8)	8.8(2.0)	69.1(1.6)	68.4(1.2)
CONST	0.1(0.1)	20.4(1.2)	0.1(0.1)	0.2(0.5)	73.0(1.0)	72.5(1.0)
PROP	24.5(1.9)	26.5(1.0)	12.0(2.3)	9.9(2.5)	70.4(1.8)	71.2(1.9)
TOPO	15.4(1.0)	0.0(0.0)	7.8(2.3)	6.3(1.8)	66.0(0.9)	66.8(1.6)
Average	15.1(0.9)	17.3(1.0)	7.4(1.6)	6.3(1.7)	69.6(1.3)	69.7(1.4)

Outside of parentheses represent the mean of the respective performance measure, while Inside of parentheses correspond to the standard deviation across the 10 times of 3-fold cross validations.

**Table 4:** Statistics values of specificity (%).

Descriptor	SVM	unSVM	Bagging	unBagging	asBagging	PRIFEAB
BCUT	99.6(0.1)	99.3(0.1)	99.2(0.1)	99.6(0.1)	68.3(0.4)	67.3(0.2)
CONST	99.9(0.1)	99.7(0.1)	99.9(0.1)	99.9(0.1)	65.2(0.5)	66.1(0.3)
PROP	99.5(0.1)	99.1(0.1)	99.3(0.1)	99.4(0.2)	70.3(0.4)	70.8(0.2)
TOPO	99.3(0.1)	100.0(0.0)	98.6(0.3)	99.2(0.1)	67.7(0.3)	67.7(0.3)
Average	99.6(0.1)	99.5(0.1)	99.3(0.1)	99.5(0.1)	67.9(0.4)	68.0(0.3)

Outside of parentheses represent the mean of the respective performance measure, while Inside of parentheses correspond to the standard deviation across the 10 times of 3-fold cross validations.

**Table 5:** Statistics values of positive predictive value (PPV) (%).

Descriptor	SVM	unSVM	Bagging	unBagging	asBagging	PRIFEAB
BCUT	50.4(2.6)	38.6(2.8)	19.1(2.7)	27.0(4.1)	3.9(0.1)	3.8(0.1)
CONST	NaN(NaN)	60.7(3.6)	NaN(NaN)	NaN(NaN)	3.8(0.1)	3.9(0.1)
PROP	4.6(0.2)	3.6(1.3)	23.2(2.2)	25.7(5.4)	4.3(0.1)	4.4(0.1)
TOPO	3.0(1.8)	NaN(NaN)	9.7(1.5)	12.8(4.2)	3.7(0.1)	3.7(0.1)
Average	19.3(1.5)	34.3(2.6)	17.3(2.1)	21.8(4.6)	3.9(0.1)	4.0(0.1)

Outside of parentheses represent the mean of the respective performance measure, while Inside of parentheses correspond to the standard deviation across the 10 times of 3-fold cross validations.

**Table 6:** Statistics values of negative predictive value (NPV) (%).

Descriptor	SVM	unSVM	Bagging	unBagging	asBagging	PRIFEAB
BCUT	98.5(0.1)	98.6(0.1)	98.3(0.1)	98.3(0.1)	99.2(0.1)	99.1(0.1)
CONST	98.2(0.1)	98.5(0.1)	98.2(0.1)	98.2(0.1)	99.2(0.1)	99.2(0.1)
PROP	98.5(0.3)	98.6(0.1)	98.4(0.1)	98.3(0.1)	99.2(0.1)	99.2(0.1)
TOPO	98.4(0.1)	98.2(0.0)	98.3(0.1)	98.3(0.1)	99.1(0.1)	99.1(0.1)
Average	98.4(0.1)	98.5(0.1)	98.3(0.1)	98.3(0.1)	99.2(0.1)	99.2(0.1)

Outside of parentheses represent the mean of the respective performance measure, while Inside of parentheses correspond to the standard deviation across the 10 times of 3-fold cross validations.

**Table 7:** Statistics values of correction (%).

Descriptor	SVM	unSVM	Bagging	unBagging	asBagging	PRIFEAB
BCUT	98.2(0.1)	97.9(0.1)	97.6(0.1)	97.8(0.1)	68.3(0.4)	67.3(0.2)
CONST	98.2(0.1)	98.3(0.1)	98.2(0.1)	98.2(0.1)	65.3(0.5)	66.2(0.3)
PROP	98.1(0.1)	97.8(0.1)	97.6(0.1)	97.8(0.1)	70.3(0.3)	70.8(0.2)
TOPO	97.8(0.1)	98.2(0.0)	97.0(0.2)	97.5(0.1)	67.6(0.3)	67.7(0.3)
Average	98.1(0.1)	98.1(0.1)	97.6(0.1)	97.8(0.1)	67.9(0.4)	68.0(0.3)

Outside of parentheses represent the mean of the respective performance measure, while Inside of parentheses correspond to the standard deviation across the 10 times of 3-fold cross validations.

**Table 8:** Statistics ratio values of the number of features used in PRIFEAB to the total number (%).

BCUT	CONST	PROP	TOPO	Average
93.3(2.0)	95.9(2.2)	98.2(0.5)	99.0(0.1)	96.6(1.2)

Outside of parentheses represent the mean value, while Inside of parentheses correspond to the standard deviation across the 10 times of 3-fold cross validations.

(1) unSVM obtains a slight improvement of ordinary SVM on three descriptors in terms of the AUC and BACC measures.

(2) Ordinary Bagging fails to improve single learning methods, not only Bagging but also unBagging get worse results than SVM and unSVM on the measures of AUC, BACC and sensitivity.

(3) asBagging and PRIFEAB obtain 20% better results than SVM, unSVM, Bagging and unBagging on the AUC measure. The sensitivity values of asBagging and PRIFEAB increase by beyond 50% from SVM, unSVM, Bagging and unBagging on average.

(4) PRIFEAB obtains slightly better results than asBagging on both sensitivity and specificity measures. We also observed that only few features are removed by feature selection.

(5) There are several cases, the learning machines fail in prediction and nearly all the examples are classified into negative, i.e. SVM, Bagging, unBagging on CONST and unSVM on TOPO. Only asBagging and PRIFEAB succeed in all predication.

## Discussions

The above results show that asBagging and PRIFEAB perform better than the other several methods of SVM, unSVM, Bagging and unBagging. Here we give some insights on these results:

(1) Though single SVM is not stable, and can not obtain valuable results, in this case of high skew data sets, bagging does not improve its generalization performance in terms of AUC, BACC and sensitivity. Bagging gets a high correction value, which is trivial, because few positive examples are predicted correctly. Especially, when learning machines fail in prediction on some descriptor data sets, all the labels are predicted as negative, a high value of correction is obtained as 98.15%, which is the ratio of negative sample to the whole sample.

(2) Since this is a drug discovery problem, we pay more attention to positives. AUC, BACC and sensitivity are more valuable than correction to measure a classifier. Asymmetric bagging and PRIFEAB improve the AUC values of ordinary bagging. Simultaneously, sensitivity are improved greatly, which shows asymmetric bagging is proper to solve the unbalanced drug discovery problem. Asymmetric bagging wins in two aspects, one is that it make the individual data subsets balanced, the second is that it pay more attention to the positives by leaving the positives always in the data set, which makes sensitivity is higher than ordinary bagging.

(3) PRIFEAB achieves slightly better results than asymmetric bagging does. Feature selection using prediction risk as criteria also make PRIFEAB win in two aspects, one is that embedded feature selection is dependent with the used learning machine, it will select features which benefit the generalization performance of individual classifiers, the second is that different features selected for different individual data subsets, which makes more diversity of bagging and improves their whole performance. The results improved by PRIFEAB than asymmetric are slight, we consider the reason is that few features are removed. Feature selection using prediction risk is dependent on SVM. Here, positives are few, which will hurt generalization performance of SVM, and furthermore hurt effect of feature selection.

(4) The data set used is so skew that the ratio of positives to negatives is only 0.0188, not beyond 2%, which makes SVM, unSVM and Baging, unBagging disable of prediction, they fail on four out of sixteen cases and predict almost all labels to negative, even on other twelve cases, they give low sensitivity. Analysis of high skew data set is still a difficult problem.

## Conclusion

To address the unbalanced problem of drug discovery, we propose to apply asymmetric bagging and feature selection to the modeling of QSAR of drug molecules. Asymmetrical bagging of SVM and a novel algorithm PRIFEAB are compared with ordinary bagging of support vector machines on a large drug molecular activities data set, experiments show that asymmetric bagging and feature selection can improve the prediction ability of SVM in terms of AUC and sensitivity. Since this is a drug discovery problem, the positive sample is few but important, AUC and sensitivity is more proper than correction to measure generalization performance of classifiers.

This work introduces asymmetric bagging into prediction of drug activities and furthermore extends feature selection to asymmetric bagging. This work only concerns an embedded feature selection model with the prediction risk criteria, one of the future work will try more efficient and more effective feature selection methods for this task.

## Methods

### Support vector machines

Support vector machines (SVM) [27] proposed by Vapnik and his co-workers in 1990s, have been developed quickly during the last decade [28], and successfully applied to biological data mining [29], drug discovery [6,8] etc. Denoting the training sample as *S *= {(**x**, **y**)} ⊆ {ℝ^*n *^× {-1, 1}}^ℓ^, SVM discriminant hyperplane can be written as

*y *= sgn(⟨**w**·**x**⟩ + *b*)

where **w **is a weight vector, *b *is a bias. According to the generalization bound in statistical learning theory [30], we need to minimize the following objective function for a 2-norm soft margin version of SVM

(1) $$\begin{array}{ll} {\text{minimize}}_{w,b} & \langle w\cdot w\rangle +C\Sigma_{i=1}^{\ell }\xi_{i}^{2}\\ \text{subject to} & y_{i}(\langle w\cdot x_{i}\rangle +b)\geq 1-\xi_{i},i=1,...,\ell , \end{array}$$

in which, slack variable *ξ*_*i *_is introduced when the problem is infeasible. The constant *C *> 0 is a penalty parameter, a larger *C *corresponds to assigning a larger penalty to errors.

By building a Lagrangian and using the Karush-Kuhn-Tucker (KKT) complementarity conditions [31,32], we can obtain the value of optimization problem (1). Because of the KKT conditions, only those Lagrangian multipliers, *α*_*i*_s, which make the constraint active are non zeros, we denote these points corresponding to the non zero *α*_*i*_s as *support vectors *(sv). Therefore we can describe the classification hyperplane in terms of *α *and *b*:

$$y=\mathrm{sgn}\left(\sum_{i\in sv}\alpha_{i}\langle x_{i}\cdot x\rangle +b\right).$$

If we replace ⟨**x**_*i*_·**x**⟩ with some function *K*(**x**_*i*_, **x**), which satisfies Mercer's condition [33], then the classification function can be written as:

$$y=\mathrm{sgn}\left(\sum_{i\in sv}\alpha_{i}K\langle x_{i}\cdot x\rangle +b\right).$$

where *K*(**x**, **z**) is the known kernel function. A commonly used kernel function is the Guass kernel:

*K*(**x**, **z**) = exp(-||**x **- **z**||^2^/*σ*^2^),

which is also preferred by C.J. Lin et al. [34].

To address the unbalanced problem, *C *in Equ.(1) is separated as *C*_+ _and *C*_- _to adjust the penalties on the false negative vs. false positive, so Equ.(1) becomes:

$$\begin{array}{ll} {\text{minimize}}_{w,b} & \langle w\cdot w\rangle +C_{+}\Sigma_{i=1;y_{i}==1}^{\ell }\xi_{i}^{2}+C_{-}\Sigma_{i=1;y_{i}==-1}^{\ell }\xi_{i}^{2}\\ \text{subject to} & y_{i}(\langle w\cdot x_{i}\rangle +b)\geq 1-\xi_{i},i=1,...,\ell , \end{array}$$

where *C*_+ _is *C *and *C*_- _is balanced_bridge*C, balanced_bridge is a coefficient, it is assigned as the ratio of the number of positive examples to that of negative ones.

The SVM obtained by the above equation is named as unSVM. This is implemented in LibSVM [35], it had been used to analysis AMDE data and is effective for unbalanced data set [17].

### Asymmetric bagging

**Figure 2:**
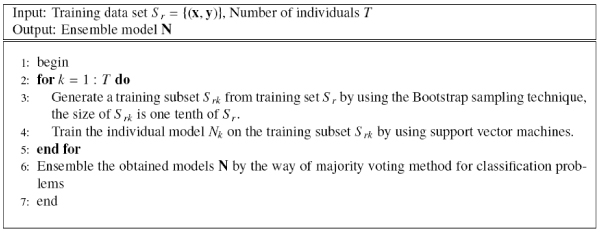
The Bagging approach.

**Figure 3:**
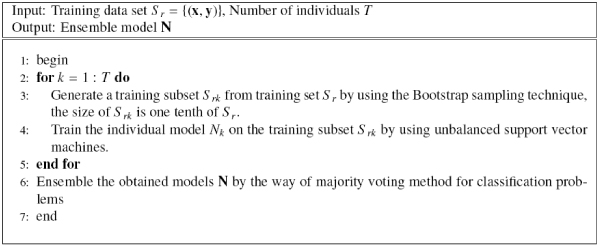
The unBagging approach.

Bagging is one of the traditional ensemble methods, which uses bootstrap to produce the diversity of individuals and uses major voting to obtain the final decision results for classification problems [10]. Figure 2 shows the ordinary bagging approach based on support vector machine. When we use a unSVM to train the individuals of bagging, we obtain the unBagging approach as in Figure 3.

**Figure 4:**
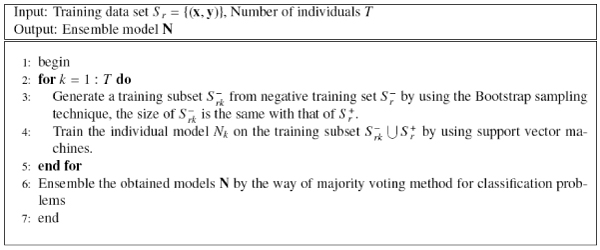
The asBagging approach.

Bagging helps to improve stable of single learning machines, but unbalance also reduce its generalization performance, therefore, we propose to employ asymmetric bagging to handle the unbalanced problem, which only execute the bootstrapping on the negative examples since there are far more negative examples than the positive ones. Tao et al. [18] applied asymmetric bagging to another unbalanced problem of relevance feedback in image retrieval and obtained satisfactory results. This way make individual classifiers of bagging be trained on a balanced number of positive and negative examples, thus solve the problem of unbalanced examples. The asymmetric bagging of SVM (asBagging) is described in Figure 4.

asBagging can solve the unstable problem of SVM classifiers and the unbalance problem in the training set.

However, it can not solve the problem of irrelevant and weak redundant features in the data sets. We will solve it by feature selection embedded in the bagging method as in the next subsection.

### PRIFEAB

Feature selection has been used in ensemble learning and obtained some interesting results, Li and Liu proposed to use the embedded feature selection method with the prediction risk criteria for bagging of SVM, where feature selection can effectively improve the accuracy of bagging methods [36]. As a feature selection method, the prediction risk criteria was proposed by Moody and Utans [37] which evaluates one feature through estimating prediction error of the data sets when the values of all examples of this feature are replaced by their mean value.

(2) $$S_{i}=\text{AUC}-\text{AUC}({\bar{x}}^{i})$$

where AUC (Area under ROC) [38] is predicted on the training data set, and AUC(${\bar{x}}^{i}$) is the prediction AUC on the training data set with the mean value of *i*^*th *^feature. Finally, the feature corresponding with the smallest will be deleted, because this feature causes the smallest error and is the least important one.

**Figure 5:**
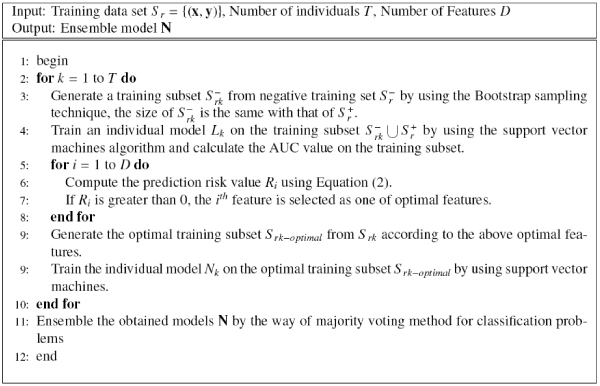
The PRIFEAB approach.

Since the asymmetric bagging method can overcome both the problems of unstable and unbalance, and feature selection can overcome the problem of irrelevant features for bagging. So we propose a novel algorithm to combine both two methods. The algorithm is named as PRIFEAB (Prediction rIsk based Feature sElection for Asymmetric Bagging), which is described in Figure 5. The basic idea of PRIFEAB is that we first use bootstrap sampling to generate a negative sample, and combine it with the whole positive sample to obtain a individual training subset. Then, prediction risk based feature selection is used to select optimal features, and we obtain an individual model by training SVM on the optimal training subset. Finally, ensemble the individual SVM classifiers by using majority voting to obtain the final model.

### NCI AntiHIV drug screen data set

The NCI AntiHIV Drug Screen data set(NCI) is obtained from . It has a categorical response measuring how a compound protects human CEM cells from HIV-1 infection. It has 29374 examples, of which 542 (1.85%) is positive and 28832 (98.15%) is negative. The structure parameters [39] consist 64 BCUT descriptors, 47 Constitutional (CONST) ones, 250 Prop ones and 266 Topological (TOPO) ones. This data set is collected and computed by Young et al., description in detail of the data set can be found in [40,41]. Here we test the proposed learning methods on individual molecular descriptors. Since the different descriptors have different meaning and few relations with each other, they can be considered as four different data sets.

### Measures

Since the class distribution of the used data set is unbalanced, only correction of classification accuracy may be misleading. Therefore, AUC (Area Under the Curve of Receiver Operating Characteristic (ROC)) [38] is used to measure the performance.

To furthermore describe the different learning methods, we also define the various measures as below [42], where *TP*, *TN*, *FP*, *FN*, stand for the number of true positive, true negative, false positive, false negative samples at classification time, respectively.

Sensitivity is defined as $\frac{TP}{TP+FN}$ and is also known as Recall.

Specificity is defined as $\frac{TN}{TN+FP}$

BACC (Balanced Accuracy) is defined as $\frac{1}{2}\left(\frac{TP}{TP+FN}+\frac{TN}{TN+FP}\right)$, which defines the average of sensitivity and specificity.

PPV (Positive Predictive Value) is defined as $\frac{TP}{TP+FP}$ and is also known as Precision.

NPV (Negative Predictive Value) is defined as $\frac{TN}{TN+FN}$.

Correction is defined as $\frac{TP+TN}{TP+TN+FP+FN}$ and measures the overall percentage of samples correctly classified.

## Competing interests

The authors declare that they have no competing interests.

## Authors' contributions

Guo-Zheng Li proposed the idea, designed the experiments and wrote the paper; Hao-Hua Meng performed experiments; Wen-Cong Lu helped in writing the paper; Mary Qu Yang helped design the experiments; Jack Y. Yang conceived and guided the project.

## References

[B1] Barrett SJ, Langdon WB (2005). Advances in the Application of Machine Learning Techniques in Drug Discovery, Design and Development. 10th Online World Conference on Soft Computing in Industrial Applications.

[B2] Tominaga Y (1999). Comparative Study of Class Data Analysis with PCA-LDA, SIMCA, PLS, ANNs, and K-NN. Chemometrics and Intelligent Laboratory Systems.

[B3] Tang K, Li T (2002). Combining PLS with GA-GP for QSAR. Chemometrics and Intelligent Laboratory Systems.

[B4] Fang KT, Yin H, Liang YZ (2004). New Approach by Kriging Models to Problems in QSAR. Journal of Chemical Information and Computer Science.

[B5] Li GZ, Yang J, Song HF, Yang SS, Lu WC, Chen NY (2004). Semiempirical Quantum Chemical Method and Artificial Neural Networks Applied for Max Computation of Some Azo Dyes. Journal of Chemical Information and Computer Science.

[B6] Xue Y, Li ZR, Yap CW, Sun LZ, Chen X, Chen YZ (2004). Effect of Molecular Descriptor Feature Selection in Support Vector Machine Classification of Pharmacokinetic and Toxicological Properties of Chemical Agents. Journal of Chemical Information & Computer Science.

[B7] Chen NY, Lu WC, Yang J, Li GZ (2004). Support Vector Machines in Chemistry.

[B8] Bhavani S, Nagargadde A, Thawani A, Sridhar V, Chandra N (2006). Substructure-Based Support Vector Machine Classifiers for Prediction of Adverse Effects in Diverse Classes of Drugs. Journal of Chemical Information and Modeling.

[B9] Dietterich T (1998). Machine-learning research: Four current directions. The AI Magazine.

[B10] Schapire R (1990). The strength of weak learn ability. Machine learning.

[B11] Breiman L (1996). Bagging predictors. Machine Learning. Machine learning.

[B12] Bauer E, Kohavi R (1999). An empirical comparison of voting classification algorithms: Bagging, Boosting, and variants. Machine learning.

[B13] Agrafiotis DK, no WC, Lobanov VS (2002). On the Use of Neural Network Ensembles in QSAR and QSPR. J Chem Inf Comput Sci.

[B14] Lanctot JK, Putta S, Lemmen C, Greene J (2003). Using Ensembles to Classify Compounds for Drug Discovery. J Chem Inf Comput Sci.

[B15] Guha R, Jurs PC (2004). Development of Linear, Ensemble, and Nonlinear Models for the Prediction andInterpretation of the Biological Activity of a Set of PDGFR Inhibitors. J Chem Inf Comput Sci.

[B16] Dutta D, Guha R, Wild D, Chen T (2007). Ensemble Feature Selection: Consistent Descriptor Subsets for Multiple QSAR Models. Journal of Chemical Information and Modeling.

[B17] Hou T, Wang J, Li Y (2007). ADME Evaluation in Drug Discovery. 8. The Prediction of Human Intestinal Absorption by a Support Vector Machine. J Chem Inf Model.

[B18] Tao D, Tang X, Li X, Wu X (2006). Asymmetric Bagging and Random Subspace for Support Vector Machines-Based Relevance Feedback in Image Retrieval. IEEE Transactions on Pattern Analysis and Machine Intelligence.

[B19] Hand DJ (1997). Construction and Assessment of Classification Rules.

[B20] Yu L, Liu H (2004). Efficient Feature Selection Via Analysis of Relevance and Redundancy. Journal of Machine Learning Research.

[B21] Kohavi R, George JH (1997). Wrappers for Feature Subset Selection. Artificial Intelligence.

[B22] Guyon I, Elisseeff A (2003). An Introduction to Variable and Feature Selection. Journal of machine learning research.

[B23] Liu Y (2004). A Comparative Study on Feature Selection Methods for Drug Discovery. J Chem Inf Comput Sci.

[B24] Li H, Yap CW, Ung CY, Xue Y, Cao ZW, Chen YZ (2005). Effect of Selection of Molecular Descriptors on the Prediction of Blood-Brain Barrier Penetrating and Nonpenetrating Agents by Statistical Learning Methods. Journal of Chemical Information and Modeling.

[B25] Eitrich T, Kless A, Druska C, Meye W, Grotendorst J (2007). Classification of Highly Unbalanced CYP450 Data of Drugs Using Cost Sensitive Machine Learning Techniques. Journal of Chemical Information and Modeling.

[B26] Li GZ, Yang J, Liu GP, Xue L (2004). Feature selection for multi-class problems using support vector machines. Lecture Notes on Artificial Intelligence 3173 (PRICAI2004).

[B27] Boser B, Guyon L, Vapnik V (1992). A Training Algorithm for Optimal Margin Classifiers. Proceedings of the Fifth Annual Workshop on Computational Learning Theory.

[B28] Cristianini N, Shawe-Taylor J (2000). An Introduction to Support Vector Machines.

[B29] Guyon I, Weston J, Barnhill S, Vapnik V (2002). Gene Selection for Cancer Classification Using Support Vector Machines. Machine Learning.

[B30] Vapnik V (1998). Statistical Learning Theory.

[B31] Karush W (1939). Minima of Functions of Several Variables with Inequalities as Side Constraints. Master's thesis.

[B32] Kuhn HW, Tucker AW (1951). Nonlinear Programming. Proceeding of the 2nd Berkeley Symposium on Mathematical Statistics and Probabilistic.

[B33] Mercer J (1909). Functions of Positive and Negative Type and their Connection with the Theory of Integral Equations. Philosophy Transactions on Royal Society in London.

[B34] Hsu CW, Chang CC, Lin CJ (2003). A Practical Guide to Support Vector Classification. Tech rep.

[B35] Chang CC, Lin CJ (2007). LIBSVM – A Library for Support Vector Machines Version 285.

[B36] Li GZ, Liu TY (2006). Feature Selection for Bagging of Support Vector Machines. PRICAI2006 Lecuture Notes in Computer Science 4099.

[B37] Moody J, Utans J, Moody JE, Hanson SJ, Lippmann RP (1992). Principled Architecture Selection for Neural Networks: Application to Corporate Bond Rating Prediction. Advances in Neural Information Processing Systems.

[B38] Duda RO, Hart PE, Stork DG (2000). Pattern Classification.

[B39] Todeschini R, Consonni V (2000). Handbook of Molecular Descriptors.

[B40] Young SS, Gombar VK, Emptage MR, Cariello NF, Lambert C (2002). Mixture Deconvolution and Analysis of Ames Mutagenicity Data. Chemometrics and Intelligent Laboratory Systems.

[B41] Feng J, Lurati L, Ouyang H, Robinson T, Wang Y, Yuan S, Young SS (2003). Predictive Toxicology: Benchmarking Molecular Descriptors and Statistical Methods. Journal of Chemical Information and Computer Science.

[B42] Levner I (2005). Feature Selection and Nearest Centroid Classification for Protein Mass Spectrometry. BMC Bioinformatics.

